# Diffuse Neurofibroma in a Micronesian Male

**DOI:** 10.7759/cureus.36542

**Published:** 2023-03-22

**Authors:** Christy Behnam, Angel Juarez, Brian Watson, Mohamed Faris

**Affiliations:** 1 Dermatology, Grand Strand Medical Center, Myrtle Beach, USA; 2 Internal Medicine, Grand Strand Medical Center, Myrtle Beach, USA; 3 Pathology, Grand Strand Medical Center, Myrtle Beach, USA

**Keywords:** neurofibroma, neurofibromatosis type 1, cafe au lait macules, von recklinghausen disease, plastic and reconstructive surgery, diffuse neurofibroma, neurofibromatosis type 1 (nf-1)

## Abstract

Neurofibromatosis type 1 (NF1) is an autosomal dominant genetic disorder, with variable clinical features, most commonly including café-au-lait macules and neurofibromas. The incidence of NF1 is approximately one in 3,000 individuals. Diffuse neurofibroma is the rarest subtype of neurofibromas. We present a case of a 39-year-old Micronesian male presenting with a substantially large and heavy overgrowth on his back, found to be consistent with diffuse neurofibroma on histopathologic examination. The patient also met the diagnostic criteria for NF1 based on clinical examination. Imaging showed the dermal and subcutaneous thickening without deep extension into the underlying fascial layer or muscles. A patient-centered, multidisciplinary approach was taken in the workup and management of this case. Our patient expressed disinterest in surgical interventions.

## Introduction

Neurofibromatosis type 1 (NF1), also known as von Recklinghausen's disease, is an autosomal dominant genetic disorder where the affected individual inherits a germline mutation in NF1, a tumor suppressor gene on chromosome 17, which codes for the neurofibromin protein. This protein helps regulate cell growth. It acts as a growth-suppressing signal to cells and regulates cell turnover through the RAS pathway. One mutation on the chromosome causes a reduction of gene function. When an additional second-hit mutation affects the NF1 gene, it renders the neurofibromin protein unable to regulate the RAS-mitogen-activated protein kinase (MAPK) pathway responsible for cell survival and proliferation [1,2].

NF1 has extensive phenotypical variability. The two cell lines most affected by NF1 mutations are Schwann cells and melanocytes. As the neurofibromin protein is unable to regulate cell growth, there is uncontrolled cell growth activity. This leads to neurofibromas and café-au-lait macules as seen in NF1. Other clinical features of NF1 include axillary and inguinal freckling, plexiform neurofibromas, nevus anemicus, juvenile xanthogranuloma, glomus tumors of the fingers and toes, iris hamartomas, and optic gliomas among others. Affected individuals may also have neurologic manifestations, such as learning difficulties, attention deficit disorder, intellectual impairment and seizures, and cardiovascular manifestations, including pulmonic stenosis, cerebrovascular anomalies, and hypertension secondary to a slightly increased risk for concurrent renal artery stenosis and pheochromocytoma. Skeletal anomalies can include scoliosis and pseudoarthrosis [1-3].

Neurofibromas are benign tumors of the peripheral nerve sheaths secondary to the proliferation of Schwann cells, perineural cells, and endoneurial fibroblasts. Neurofibromas account for approximately 5% of all benign soft tissue tumors. There are three main subtypes of neurofibroma: localized, plexiform, and diffused. The localized subtype is the most common and the plexiform subtype is pathognomonic for NF1, whereas the diffuse subtype is the rarest. When reported, diffuse neurofibromas are primarily identified in adolescents and young adults with significant dermal and subcutaneous thickening affecting the trunk, head, and neck regions. Although these individuals usually develop benign tumors, they also have a lifetime risk of malignant transformation of 5%. We report a case of a diffuse neurofibroma on the back of a 39-year-old Micronesian male presenting as substantially large and heavy overgrowth [2,3].

## Case presentation

A 39-year-old male from Micronesia with no past medical history presented with a five-day history of increased pain, swelling, and raised temperature on palpation associated with a large overgrowth on his back. He stated that it had been an enlarging birthmark over the past 10 years. The overgrowth was heavy, causing him moderate back pain that was exacerbated by movement and relieved by rest and offloading the overgrowth weight. Upon review of the systems, the patient endorsed low-grade fevers and chills, pruritus, weight gain, and joint pain, especially in the shoulders, wrists, and back. No other pertinent positive or negative symptoms were noted. The patient denied any past medical or surgical history and reported that he had not seen a healthcare provider in many years and does not take any medications at home. He does not smoke or use any illicit drugs, and he quit alcohol 10 years ago after a positive history of alcohol abuse. The patient has been able to maintain a job despite the pain and discomfort associated with the tissue burden. Family history was significant for family members who had multiple unknown overgrowths over their skin but to a much lesser extent and severity.

**Figure 1:**
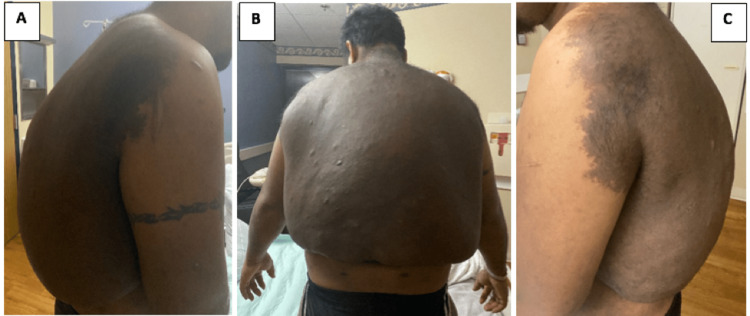
Clinical images of the large tumor overgrowth over the thoracic and lumbar back at the time of presentation showing right lateral, posterior, and left lateral views, respectively.

**Figure 2:**
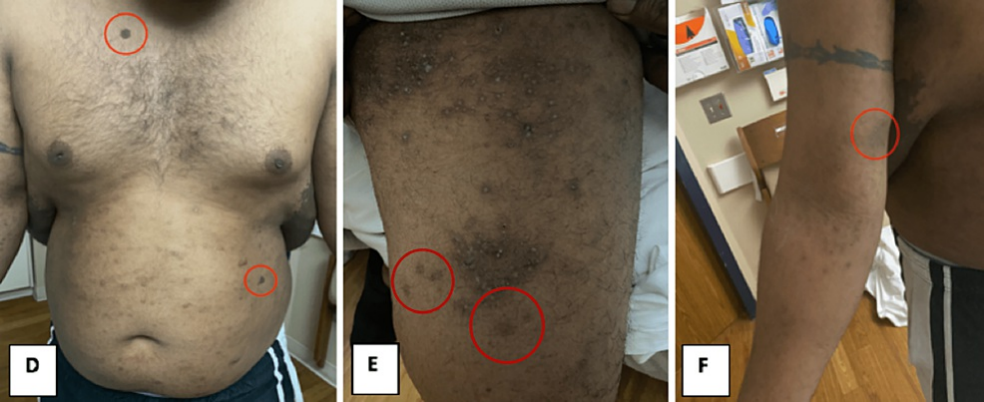
Clinical images of the anterior chest and abdomen, left thigh, and right upper extremity, respectively, with red circles highlighting the presence of a few of the patient’s large café au lait spots.

Vital signs were stable with the patient being afebrile and normotensive, and the physical exam revealed a large hyperpigmented tumor covering the thoracic and lumbar back, extending superiorly over the shoulders bilaterally and the base of the neck, with a tender superimposed area of fluctuance, noted over the right side of the midline overlying the inferior border of the tumor (Figure 1). The patient also had numerous (>6), large (>15 mm) cafe-au-lait spots (Figure 2), and a few neurofibromas scattered throughout his body. Neurologic, musculoskeletal, cardiac, respiratory, abdominal, and lymphatic exams were normal.

Laboratory testing showed leukocytosis, normocytic anemia, eosinophilia, elevated sedimentation rate and C-reactive protein, mild hyperglycemia, and mild transaminitis. The lipid panel showed elevated low-density lipoprotein but was otherwise normal. Coagulation studies, thyroid-stimulating hormone, hepatitis panel, lactic acid, and hemoglobin A1c were within normal limits (Table 1). The antinuclear antibody was negative, and complement component 3 was elevated while complement component 4 was normal. Human immunodeficiency virus testing was negative. Two blood samples were obtained for blood cultures without any growth for five days.

**Table 1:** Laboratory results. Hgb: hemoglobin; HCT: hematocrit; MCV: mean corpuscular volume; RDW: red cell distribution width; PLT: platelet; LDL: low-density lipoprotein; HDL: high-density lipoprotein; BUN: blood urea nitrogen; EST GFR: estimated glomerular filtration rate; CKD-EPI: Chronic Kidney Disease Epidemiology Collaboration; AST: aspartate aminotransferase; ALT: alanine aminotransferase; INR: international normalized ratio; PT: prothrombin time; PTT: partial thromboplastin time.

Complete blood count			Chemistry	
WBC	15.3 K/mm3 (3.7-10.1)		Sodium	137 mmol/L (136-145)
Hgb	14.1 gm/dl (14.0-16.4)		Potassium	3.5 mmol/L (3.5-5.1)
HCT	41.5% (40.0-47.2)		Chloride	106 mmol/L (98-107)
MCV	84.0 fL (81.8-94.6)		Carbon dioxide	23 mmol/L (21-32)
RDW	14.2% (11.6-14.0)		Anion gap	8.0 mEq/L (3.0-11.0)
PLT count	186 K/mm3 (150-400)		BUN	10 mg/dl (7-18)
Sedimentation rate	92 mm/hour (0-15)		Creatinine	0.9 mg/dl (0.6-1.3)
C-reactive protein	16.4 mg/dL (0-0.99)		EST GFR (CKD-EPI)	>60 (>=60)
			Glucose	131 mg/dl (74-106)
Lipid panel			Calcium	8.8 mg/dl (8.5-10.1)
Triglycerides	105 mg/dL (0.0.99)		AST	40 units/L (11-38)
Cholesterol	207 mg/dL (5--199)		ALT	63 units/L (10-47)
LDL cholesterol	114 mg/dL (0-100)		Total protein	9.0 gm/dl (6.4-8.2)
HDL cholesterol	32 mg/dL (0-40)		Albumin	3.6 gm/dl (3.5-5.0)
Hemoglobin A1c	5.3% (3.8-5.6%)		Lactic acid	1.1 mmol/L (0.7-2.1)
			Coagulation panel	
			INR	1.20 (0.9-1.1)
			PT	13.6 s (9.8-13.9)
			PTT	30.8 s (24.9-37.9)

**Figure 3:**
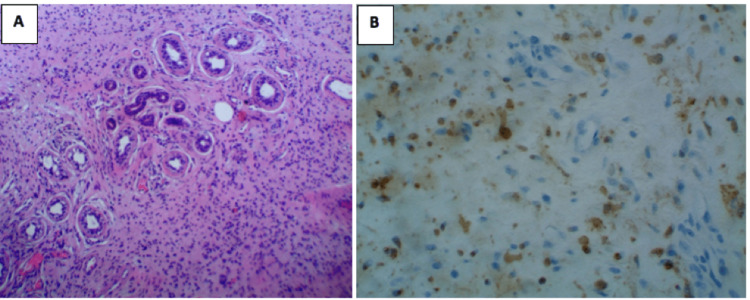
(A) Histopathologic examination showing bland spindle cell proliferation growing around skin adnexal structures on hematoxylin and eosin staining (original magnification, x10). (B) Immunohistochemical staining showing S-100 positivity (original magnification, x40).

Initial punch biopsy of a representative area on the back was indeterminate, with no malignancy identified. Two additional excisional biopsies were obtained by general surgery from the right posterior axillary line and right lateral back. Histological examination of both excisional tissue samples showed bland spindle cell proliferation growing around skin adnexal structures (Figure 3A). Immunohistochemical staining was positive for S-100 (Figure 3B) and negative for pan-keratin, cluster of differentiation 34 (CD34), melanoma-associated antigen recognized by T cells (MART-1), melan-A, and tyrosinase. A diagnosis of diffuse neurofibroma was subsequently established.

**Figure 4:**
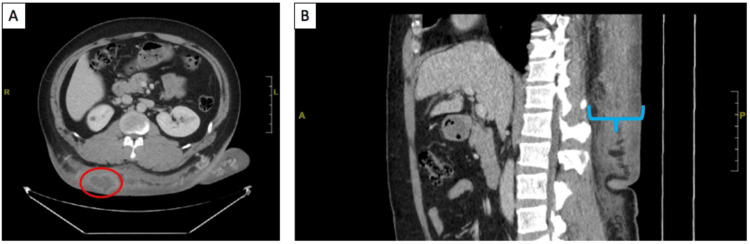
Abdominal CT. (A) Axial view. The red circle encloses the fluid collection measuring 5.0 x 3.2 x 4.0 cm. (B) Sagittal view of the patient. The blue bracket highlights the neurofibroma on the patient's back.

Multiple imaging modalities were obtained. Chest computed tomography (CT) showed diffuse abnormal soft tissue pathology seen throughout the subcutaneous tissues of the back with 5.9 x 36 x 38 cm thickness and craniocaudal and transverse length, respectively. Pathologic bilateral axillary lymphadenopathy was noted, with the largest lymph node measuring 2.5 x 1.3 cm within the right axilla. Oncology was consulted, and an ultrasound-guided left axillary lymph node core biopsy by interventional radiology showed lymphoid tissue composed of small to intermediate lymphocytes with mixed B and T cells. No lymphoma or metastatic carcinoma was identified. Abdominal CT revealed a 5.0 x 3.2 x 4.0 cm rim-enhancing fluid collection along the inferior right confines of the soft tissue mass in the back, resembling a necrotic tumor or an infected fluid collection. Ultrasound-guided aspiration of the fluid collection showed abundant neutrophils, but cytology did not detect malignant cells. Aspirated culture from the fluid collection showed no aerobic or anaerobic growth. A right upper quadrant ultrasound was normal. Magnetic resonance imaging (MRI) of the back was consistent with the CT findings, and an MRI of the neck showed preserved fascial layer and muscles with no deep extension of the skin thickening observed (Figure 4).

Due to chills and leukocytosis, the patient was started on vancomycin and ceftriaxone. The patient was continued on vancomycin but soon switched to meropenem for broad-spectrum coverage. Infectious disease was consulted, and the regimen was later deescalated to amoxicillin-clavulanic acid. Since the leukocytosis and chills eventually resolved and infectious etiologies were ruled out, antibiotics were subsequently discontinued. Associated pain was managed with a multimodal pain regimen, and pruritus was managed with oral hydroxyzine and topical triamcinolone. After the above workup and management, the patient was discharged home and instructed to follow up at our clinic. Outpatient referrals were also sent to oncology and plastic surgery for surgical debulking. The patient did follow up in our clinic and shared that he continues to have moderate back pain, associated with the heaviness of the diffuse neurofibroma, but that he is otherwise asymptomatic. The benefits of referrals and seeing other specialists were discussed again, and the patient expressed disinterest in surgical interventions and elected to forgo any of the referrals offered at this time.

## Discussion

The diagnosis of NF1 can be established using the revised diagnostic criteria (Table 2), adapted from Legius et al. [4]. In our post-pubertal patient without a family history of an NF1-diagnosed parent, the presence of six or more café-au-lait macules over 15 mm in greatest diameter in addition to the multiple neurofibromas scattered over his body established the diagnosis of NF1.

**Table 2:** Diagnostic criteria for neurofibromatosis type 1. Adapted from Legius et al. [4].

Revised diagnostic criteria for neurofibromatosis type 1 (NF1)	
A: The diagnostic criteria for NF1 are met in an individual who does not have a parent diagnosed with NF1 if two or more of the following are present:	
	Six or more café-au-lait macules over 5 mm in greatest diameter in prepubertal individuals and over 15 mm in greatest diameter in postpubertal individuals
	Freckling in the axillary or inguinal region
	Two or more neurofibromas of any type or one plexiform neurofibroma
	Optic pathway glioma
	Two or more iris Lisch nodules identified by slit lamp examination or two or more choroidal abnormalities (CAs)—defined as bright, patchy nodules imaged by optical coherence tomography (OCT)/near-infrared reflectance (NIR) imaging
	A distinctive osseous lesion such as sphenoid dysplasia, anterolateral bowing of the tibia, or pseudarthrosis of a long bone
	A heterozygous pathogenic NF1 variant with a variant allele fraction of 50% in apparently normal tissue such as white blood cells
B: A child of a parent who meets the diagnostic criteria specified in A merits a diagnosis of NF1 if one or more of the criteria in A are present	

The tumor on the back was biopsied to confirm the lesion type and guide management. The histopathologic examination findings of bland spindle cell proliferation growing around skin adnexal structures on hematoxylin and eosin staining, in addition to the cells reacting with antibodies against S100-protein on immunohistochemical staining, confirmed the diagnosis of diffuse neurofibroma. Despite the hyperpigmentation seen clinically, the negative MART-1 stain excludes classifying this diffuse neurofibroma as melanotic. This distinction is crucial as there have been reported cases of melanotic plexiform neurofibroma [5]. Imaging identified a rim-enhancing fluid collection, raising concerns regarding a necrotic tumor or an infected fluid collection. However, since the cultures of the aspirates ruled out any infectious etiologies, the transient leukocytosis, low-grade fever, and chills were attributed to be secondary to inflammation caused by tumor cell necrosis outgrowing their blood supply due to uncontrolled proliferation.

Early presentation and seeking medical attention promptly are of paramount importance in all medical diseases, especially regarding diffuse neurofibroma growths. Delayed presentation in the magnitude of decades as is the case in our patient presentation, unfortunately, allowed the once small overgrowth to enlarge extensively to a degree where it became considerably substantial and heavy, causing discomfort, pain, and disfiguration. Carrying the weight of the diffuse neurofibroma strained the back of our patient and resulted in chronic back pain.

Management of individuals with NF1 requires a patient-centered, multidisciplinary approach. Referring the patient for genetic counseling can be considered to confirm the diagnosis and provide further counseling regarding this autosomal dominant disease. Our patient was not interested in genetic counseling, and as such, genetic testing to confirm the NF1 mutation was not performed. Surgical consultation is often required for large neurofibromas that can be especially disfiguring or painful. Our patient was referred to plastic surgery consultation for excising the large, painful, heavy, and disfiguring diffuse neurofibroma on his back. However, the patient was not interested in any surgical intervention and elected to forgo the surgery consultation. An oncology referral was also provided to support any additional recommended testing and further monitoring modalities, but the patient shared that he does not intend on following up with oncology. Our patient did not have neurological or ophthalmological abnormalities on a thorough history and neurological and ophthalmological examination; therefore, neurological imaging modalities were unnecessary. Similarly, our patient was normotensive; therefore, a complete cardiac assessment was not indicated, and renal arteriography and 24-hour urine collection for catecholamines and metanephrines to rule out concurrent renal artery stenosis and pheochromocytoma, respectively, were not necessary [1]. Given that our patient was an adult at the time of diagnosis and lacked any evidence of scoliosis on examination, serial exams performed annually to evaluate the progression of scoliosis were not applicable [1].

## Conclusions

Ultimately, we present a rare case of a young Micronesian male with a substantially large and heavy diffuse neurofibroma, in the setting of a concurrently diagnosed NF1. Although a rare subtype of benign tumors of the peripheral nerve sheets, diffuse neurofibroma can have tremendous growth leading to discomfort, pain, and disfigurement. We shed light on exacerbated severity of growth size associated with delayed presentation. We also highlight the importance of a patient-centered, multidisciplinary approach in managing patients with this disorder.
